# Effects of repeated tDCS on lower limb muscle explosive strength and executive function in male collegiate volleyball players: a randomized controlled trial

**DOI:** 10.3389/fphys.2026.1717237

**Published:** 2026-04-10

**Authors:** Meichen Liu, Bing Han, Yuning Sun, Chunhui Yang

**Affiliations:** Department of Sports, Northeastern University, Shenyang, China

**Keywords:** electroencephalography (EEG), executive function, lower limb explosive strength, male collegiate volleyball players, repeated tDCS

## Abstract

**Objective:**

Volleyball requires superior lower limb explosive strength and rapid executive function for optimal performance and injury risk reduction, yet the efficacy of repeated transcranial direct current stimulation (tDCS) in enhancing these volleyball-specific physical and cognitive capacities remains unclear. This study aimed to investigate the effects of five consecutive days of tDCS on lower limb explosive strength and executive function in male collegiate volleyball players.

**Methods:**

Thirty-two participants were randomized to receive real tDCS (anodal stimulation over M1, 2 mA for 20 min/day, n = 17) or sham stimulation (n = 15) for five consecutive days. Running jump height, countermovement jump (CMJ) height, isokinetic knee strength at 60°/s and 180°/s (extension/flexion, dominant/non-dominant leg), and event-related potential (ERP) indices (No-Go P3 amplitude/latency) during a Go/No-Go task, as well as reaction time (RT) in the task were assessed before and after the intervention.

**Results:**

A significant Time × Group interaction was observed for running jump height (*P* < 0.01) and No-Go P3 amplitude (*P* < 0.01), with the tDCS group showing greater improvements than the sham group. For CMJ, isokinetic knee extensor strength (60°/s) and RT, no significant Time × Group interaction was observed, while a significant main effect of Time was detected (all *P*s < 0.05) without significant interaction. No-Go P3 latency showed no significant effects.

**Conclusion:**

Five consecutive days of tDCS on the M1 area specifically enhances volleyball-related explosive jumping performance (running jump) and inhibitory control (increased Nogo-P3 amplitude) in male collegiate volleyball athletes.

## Introduction

Volleyball is a sport that demands high levels of skill and explosive power, particularly in the lower limb muscles. Frequent jumping, blocking, and spiking movements in volleyball place stringent demands on the lower limb strength and explosive power of athletes. Lower limb explosive power not only directly influences athletic performance (Novita et al., 2022) but is also closely associated with the risk of injuries (Guirelli et al., 2021). Studies have shown that the leg muscle strength of volleyball players significantly impacts their performance during matches, particularly in spiking and blocking. Athletes with greater muscle strength can execute spikes and blocks at greater heights, thereby gaining a tactical advantage (Reitmayer and Monea, 2021; Novita et al., 2022). Additionally, insufficient dynamic stability not only affects athletic performance but also increases the risk of muscle injuries. Weak lower limb strength may lead to severe knee and ankle injuries, whereas stronger muscle strength can enhance stability and reduce the incidence of injuries (Guirelli et al., 2021).

Volleyball is a dynamic sport requiring rapid decision-making and precise motor control, which heavily relies on executive functions such as inhibitory control, working memory, and cognitive flexibility. These functions enable athletes to suppress irrelevant responses, maintain task goals, and adapt to unpredictable game situations (Alves et al., 2013; Montuori et al., 2019; Formenti et al., 2021). Research by Alves et al. (2013) found that elite volleyball players exhibit superior inhibitory control when performing complex tasks, which helps them better eliminate interference, stabilize neuromuscular activation patterns, reduce unnecessary movement deviations, and execute actions more fluidly and precisely. Therefore, improving inhibitory control is crucial for volleyball athletes. The inhibitory control—reflected in the event-related potential (ERP) component No-Go P3 is critical for filtering distractions and executing timely actions during matches (Falkenstein, 2006; Guo et al., 2015). No-Go P3, a positive ERP component peaking at 300–600 ms during No-Go trials, is a core neurophysiological marker of response inhibition and cognitive evaluation processes in the fronto-parietal network (Falkenstein et al., 1999), and its amplitude directly indexes the efficiency of inhibitory control and task processing.

Transcranial direct current stimulation (tDCS), a non-invasive brain stimulation technique, delivers weak direct current through electrodes placed on the scalp to modulate cortical excitability. Anodal stimulation increases cortical excitability, while cathodal stimulation decreases it (Nitsche and Paulus, 2000; Vergallito et al., 2023). The application of tDCS in the field of sports has become increasingly widespread, particularly in enhancing muscle strength and explosive power. Studies have shown that tDCS can improve athletic performance by increasing corticospinal excitability or optimizing muscle recruitment strategies (Kamali et al., 2019; Kristiansen et al., 2022). Hadi Fahrvandi et al. (2023) investigated the effects of tDCS on countermovement jump (CMJ) performance in non-elite jumpers, which indicated that tDCS had no significant impact on maximal force, time to reach maximal force, or rate of force development, suggesting that a single session of anodal tDCS may be insufficient to significantly enhance jumping performance. In contrast, Kamali et al. (2019) found that tDCS applied to the primary motor cortex significantly improved muscle strength in experienced bodybuilders. However, Machado et al. (2021) reported that neither high-definition tDCS (HD-tDCS) nor conventional tDCS significantly altered endurance performance in endurance athletes. A systematic review suggested that tDCS may have a more pronounced effect on improving strength than on endurance performance (MaChado et al., 2019b). Overall, the findings on the effects of tDCS on muscle strength are inconsistent, which may be attributed to variations in stimulation parameters such as dosage, current intensity, target brain regions, and stimulation duration (MaChado et al., 2019a; da Silva MaChado et al., 2021). Regarding tDCS dosage, single-session tDCS appears to have limited effects on muscle strength. Studies have shown that a single session of tDCS does not significantly enhance muscle strength (MaChado et al., 2019a; Mesquita et al., 2020; Zhang et al., 2025). The effects of multiple tDCS sessions on muscle strength remain unclear, as there are few studies on repeated tDCS interventions, and the results are inconsistent (Chinzara et al., 2022). Repeated stimulation over consecutive days is thought to promote long-term potentiation-like plasticity, leading to more stable behavioral improvements (Reis et al., 2009). The 5-day consecutive protocol has been established as an effective and replicable paradigm in prior tDCS studies targeting motor learning and performance enhancement (Reis et al., 2009). Therefore, this study aims to investigate the effects of multiple tDCS sessions on lower limb muscle strength in male collegiate volleyball players.

In addition to its effects on muscle strength, tDCS has also been shown to modulate executive functions by enhancing cortical excitability (Nitsche and Paulus, 2000). Previous studies suggest that repeated tDCS can improve inhibitory control, as evidenced by increased No-Go P3 amplitudes (Leite et al., 2011; Moezzi et al., 2023). However, some studies have reported no significant cognitive enhancement from tDCS. Seidel and Ragert (2019) compared the effects of tDCS on reaction time and tapping tasks in trained football and handball athletes versus non-athletes, finding no significant improvement in performance (Seidel and Ragert, 2019). Moreira et al. (2023) investigated the effects of a single session of tDCS on professional female basketball players during prolonged cognitive tasks and found that tDCS did not significantly mitigate the negative impact of cognitive fatigue on technical performance (Moreira et al., 2023). Grosprêtre et al. (2021) studied the effects of a single session of tDCS on parkour athletes and reported that anodal tDCS applied to the primary motor cortex and left dorsolateral prefrontal cortex (dlPFC) improved jumping performance but had no significant effect on cognitive tests (Grosprêtre et al., 2021). These inconsistencies may be attributed to dosage parameters.

This study investigates whether five consecutive days of anodal tDCS over the primary motor cortex (M1) can enhance lower limb explosive strength and executive functions in male collegiate volleyball players, as measured by strength indices (countermovement jump, running jump, and isokinetic strength of knee joint) and ERP indices (No-Go P3 amplitude/latency) during a Go/No-Go task. We hypothesized that five consecutive days of anodal tDCS over the primary motor cortex (M1) would specifically enhance volleyball-related explosive jumping performance, particularly the running jump which closely mimics sport-specific actions, and improve executive function, as reflected by increased No-Go P3 amplitude (indicating stronger response inhibition efficiency), while reducing No-Go P3 latency.

## Materials and methods 

### Participants

Sample size was calculated using GPower 3.1.9.7 for a 2×2 repeated measures ANOVA, with an effect size f = 0.45 [based on prior tDCS studies in athletes of Lattari et al. (2020)], α = 0.05, and power = 0.80). This yielded n=14 participants/group. Initially, 18 participants were recruited for each group, divided into an experimental group (tDCS) and a control group (Sham) (**Table 1**). However, due to factors such as training commitments, 4 participants withdrew from the study (1 in the tDCS group and 3 in the sham group). At last, this study recruited 32 male collegiate volleyball players as participants. All participants were required to have a certain level of training experience and skill proficiency, specifically at the national second-grade athlete level. The age range of the participants was between 18 and 25 years old, with no history of severe neurological or musculoskeletal disorders. Additionally, participants should not have undergone any other forms of motor skill learning interventions. Inclusion Criteria: (1) Aged between 18 and 25 years; (2) Possess at least three years of volleyball training experience and hold a national second-grade athlete certification; (3) No history of severe neurological or musculoskeletal disorders. Exclusion Criteria: (1) History of allergic reactions (e.g., contact dermatitis, severe skin irritation) to electrode materials, conductive gels, or skin preparation products used in transcranial electrical stimulation; (2) Athletes with lower extremity disorders, such as pain, injury, or surgery within the last six months, were excluded; (3) Failure to meet the inclusion criteria. Each subject was informed about the experimental process and signed an informed consent form and the Ethics Committee of this University approved this study.

**Table 1:** Demographic characteristic of participants.

Variables	tDCS (n = 17)	Sham (n = 15)	t value	*P* value
Age (years)	19.8 ± 1.8	19.6 ± 1.9	0.242	0.811
Height (cm)	181.9 ± 5.6	178.8 ± 5.9	1.513	0.141
Weight (kg)	78.3 ± 8.0	72.8 ± 12.2	1.475	0.153
Body mass index (kg/m^2^)	23.7 ± 2.3	22.8 ± 4.1	0.671	0.509
Years of experience (years)	3.7 ± 1.1	3.2 ± 1.1	1.222	0.231
Vertical jump height (cm)	63.3 ± 9.3	64.4 ± 5.6	0.734	0.468

### Experimental design

This study adopted a randomized, single-blind, placebo-controlled experimental design. Blinding integrity was assessed post-intervention via a 5-point Likert scale questionnaire (1 = “certainly sham” to 5 = “certainly real tDCS”) administered to participants. Outcome evaluators (assessing jump performance, isokinetic strength, and EEG data) were kept unaware of group assignments throughout the entire process of data collection and processing. For the participant self-assessment of stimulation type, the mean Likert scale rating was 2.9 ± 0.8 for the tDCS group and 3.1 ± 0.7 for the sham group; a formal statistical assessment of correct versus incorrect group allocation guesses was not performed in this study, and the above rating results only reflect the participants’ subjective uncertainty about their intervention group. Participants were required to undergo 20 minutes of tDCS prior to exercise training, followed by regular training sessions, for five consecutive days. Both groups maintained identical training volume and intensity. Baseline measurements were conducted before the experiment, including assessments of lower limb explosive power and reaction time in the Go/No-Go task, and the amplitude and latency of the No-Go P3 component. After the 5-day intervention, the same measurements were repeated, and the pre- and post-intervention data were compared and analyzed.

### Instruments and equipment

The instruments and equipment used in this study included a Transcranial Direct Current Stimulation (tDCS) device (Halo Sport, USA), an isokinetic dynamometer (ISOMED2000, Germany), an electroencephalography (EEG) system (eego, ANT, Germany), and EEG conductive gel.

### Experimental procedure

Prior to the main experiment, baseline assessments were conducted for all participants to evaluate their lower limb muscle strength, explosive power, and cognitive function. These baseline tests were completed within one week. The experimental group received 20-minute tDCS sessions at 2 mA, while the control group received sham stimulation. The intervention was administered for five consecutive days. After the 5-day intervention, all participants underwent post-intervention assessments of the same metrics. Cognitive performance assessments were conducted first, followed by lower limb strength measurements.

### tDCS protocol

In this study, a portable transcranial direct current stimulation (tDCS) device (specifically the Halo Sport) was employed with a current intensity of 2 mA and a stimulation duration of 20 minutes. The Halo Sport device was properly aligned on the participant’s cranium to ensure optimal neural stimulation. Preconfigured electrode placement targeting the M1 leg representation, which is anatomically aligned with the international 10–20 system and validated in prior motor performance studies (Huang et al., 2019; Lu et al., 2021). Prior to application, three saline-soaked sponge electrodes (each 6.4 cm × 4.4 cm, total contact area ≈ 28 cm² per electrode; Halo Sport standard specification) were prepared using 0.9% sodium chloride solutions to establish reliable electrical conductivity with the scalp. Based on the protocols described by Lu et al. (2021) and Codella et al. (2021), the electrodes were strategically positioned over the central and bilateral leg areas of the primary motor cortex (M1), intersecting the vertex region. The anode was placed at the Cz electrode site (primary motor cortex leg area) (Jurcak et al., 2007; Lu et al., 2021), while the cathodes were symmetrically positioned at C5 and C6 according to the international 10–20 system, thereby enabling bilateral tDCS of the motor cortex. For the experimental group, the tDCS current intensity gradually increased to 2 mA over 30 seconds and was maintained at this level for the remainder of the 20-minute session. Sham stimulation included 30-second current ramping at the start/end to mimic real stimulation, with no current delivered during the 20-minute session, serving as a placebo control (Lu et al., 2021). In our study, the training regimen commenced exactly 5 minutes after the completion of the 20-minute tDCS stimulation. This 5-minute interval was chosen based on prior studies indicating that anodal tDCS-induced increases in cortical excitability begin shortly after stimulation onset and are sustained for a period thereafter (Nitsche and Paulus, 2001).

### Testing and evaluation of lower limb muscle strength

#### Isokinetic muscle strength

This study utilized an isokinetic dynamometer to assess the muscle strength of the bilateral knee joints at different movement velocities. Participants performed a thorough warm-up, including 5–10 minutes of low-intensity aerobic exercise (e.g., treadmill or cycling) and dynamic stretching, to prepare their muscles and joints. Following the warm-up, participants completed familiarization trials on the isokinetic dynamometer to ensure they were comfortable with the equipment and testing procedures.

Before testing, the isokinetic dynamometer was calibrated and adjusted to fit the participant’s body dimensions and knee joint angles. Participants were seated on the dynamometer with their back firmly against the seat, hips and knees fixed at 90° and 70°, respectively, and ankles secured to the footplate. Straps were tightened to ensure stability without restricting blood circulation. During testing, participants held the side handles of the seat to maintain stability.

The dynamometer was set to velocities of 60°/s and 180°/s (Wilkosz et al., 2021; Berriel et al., 2024). According to Wilkosz et al. (2021), prior to formal testing, participants performed several low-intensity practice trials to familiarize themselves with the equipment and procedures. During the formal test, participants completed five consecutive maximal concentric knee extension and flexion efforts at each velocity. Participants were instructed to exert maximal force throughout the movement and maintain peak effort until the end of each trial. Researchers provided verbal encouragement to ensure maximal effort. At each angular velocity (60°/s and 180°/s), participants completed 1 set of 5 maximal repetitions for knee flexion and extension. To prevent fatigue, adequate rest was provided between tests at 60°/s and 180°/s, as well as between dominant and non-dominant leg assessments. To ensure counterbalancing, the order of tests was randomized.

#### Countermovement jump, running jump, and standing long jump tests

1. Countermovement Jump: the participant started from an upright standing position on a force plate (Kistler, Swiss), performed a quick knee flexion (countermovement) while simultaneously swinging the arms backward, and then explosively extends the knees and hips while swinging the arms forward to achieve maximal vertical height (Petrigna et al., 2025). Each participant completed three trials, and the highest jump was calculated.
2. Running Jump: participants executed a running approach followed by a two-footed takeoff, utilizing arm swing and knee flexion to achieve maximal vertical height by an electronic touch height sensor (Kakihana and Suzuki, 2001). Three trials were performed, and the highest jump was recorded.
3. Standing Long Jump: Participants stood behind the starting line with feet shoulder-width apart, swung their arms backward, and bent their knees before explosively jumping forward. The distance from the starting line to the heel of the participant’s landing position was measured (Marin-Jimenez et al., 2024). Three trials were conducted, and the longest jump was recorded.

To avoid order effects, the sequence of tests was randomized and counterbalanced. All tests for a single participant were completed on the same day.

### Inhibitory control assessment via EEG during the Go/No-Go task

This study employed a visual Go/No-Go paradigm to assess inhibitory control via EEG (Cheng et al., 2019). Participants were seated in a quiet, moderately lit laboratory room on a comfortable chair and instructed to remain relaxed. After washing their hair, participants were fitted with an EEG cap, and conductive gel was applied to ensure all electrode impedances were kept below 5 kΩ. Prior to the formal experiment, a brief practice session consisting of 10–20 stimuli was conducted to familiarize participants with the Go/No-Go task. During the practice session, feedback such as “You pressed correctly” or “You should inhibit this response” was presented as text on the computer screen via the E-prime 3.0 stimulus presentation software to participants.

In the formal experiment, participants were presented with a series of randomly displayed visual stimuli, including a green circle and a green triangle (Chen and Watanabe, 2023). Participants were instructed to respond quickly when the green circle appeared and to withhold their response when the green triangle appeared. The two types of stimuli were presented in equal proportions (50% each) (Zhang et al., 2024), with a total of 150 stimuli presented randomly. Each stimulus was displayed for 100 milliseconds, and the inter-stimulus interval varied randomly between 1000 and 1500 milliseconds (i.e., it could be any value within this range) to avoid anticipation effects (Cheng et al., 2019). Participants were instructed to press a response button as quickly and accurately as possible when the target stimulus (green circle) appeared. Throughout the experiment, participants were required to maintain focus, minimize blinking, and avoid head movements to reduce motion artifacts in the EEG signals. Reaction times for correct responses were recorded and analyzed.

### Data processing and analysis

#### Data processing and analysis of muscle strength

For isokinetic muscle strength assessment, the peak torque during five consecutive flexion-extension movements of the knee joint in both the dominant and non-dominant limbs was selected as the observational index.

#### EEG signal acquisition and processing

In this study, a portable 32-channel EEG device was used to collect the EEG signals of participants while they were performing the Go/No-Go task. The signal sampling frequency was 1000 Hz, the band-pass filtering range was 0.1–100 Hz, and the reference electrode was at the Pz point.

To extract the No-Go P3 component in the Go/No-Go paradigm, preprocessing of the EEG data was required first. After the experiment, the EEG data was first imported into EEGLAB. Firstly, the data was subjected to band-pass filtering between 0.1 Hz and 30 Hz to remove the DC drift and high-frequency noise. Then, the continuous EEG signal was segmented into event-related time epochs, from 100 milliseconds before the stimulus to 800 milliseconds after the stimulus. A baseline correction was performed for each epoch, with the 100 ms pre-stimulus interval serving as the baseline period; the mean voltage of this pre-stimulus baseline was subtracted from all time points within the epoch to eliminate the influence of spontaneous cortical activity and chronic potential drift. Next, artifact correction was carried out through independent component analysis (ICA). After separating the independent components, the components related to artifacts such as eye movements and blinks were identified and removed. After artifact correction, the data was re-referenced to the average reference to reduce the noise of the reference electrode itself. Subsequently, artifact rejection was performed again, and the time epochs with amplitudes exceeding the set threshold (± 80 μV) were excluded. Event-related potential (ERP) components were extracted by averaging the artifact-corrected trials of the same type to improve the signal-to-noise ratio. For artifact rejection, the average number of epochs rejected per participant was 13.1 ± 3.2 in the tDCS group and 11.4 ± 3.6 in the sham group, and independent samples t-test confirmed no statistically significant difference in the number of rejected epochs between the two groups (*P* > 0.05). In this study, the P3 component under the No-Go condition was extracted, and the time window was the positive-going wave between 300–600 milliseconds after the stimulus (Polich, 2007). For the peak latency, No-Go P3 latency was the time point (ms) of the most positive voltage within the 300~600 ms window, both relative to stimulus onset (0 ms). For the peak amplitude, No-Go P3 amplitude was the most positive voltage at the identified P3 peak latency within the 300~600 ms window. We performed a topographic analysis of the No-Go P3 component across all electrode sites. For each participant, we identified the peak amplitude of the No-Go P3 within the 300–600 ms time window, and then extracted the amplitude values at that time window from all scalp electrodes. Grand-averaged topographic maps were constructed based on these peak amplitudes (Polich et al., 1997). Finally, the Cz electrode position was selected for the No-Go P3 amplitude analysis.

### Statistical analysis

Statistical analyses were performed using SPSS 19.0 and GraphPad Prism 9.0 for data processing and visualization. A 2×2 mixed-design analysis of variance (ANOVA) was employed to examine the effects of tDCS on lower limb muscle strength and cognitive performance in male volleyball players. The independent variables included one within-subjects factor: intervention time (two levels: pre-intervention and post-intervention), and one between-subjects factor: intervention method (two levels: tDCS and sham). The dependent variables included knee joint explosive power, reaction time, and the amplitude and latency of the No-Go P3 component. Normality tests confirmed that all dependent variable data followed a normal distribution. Data are presented as mean ± standard deviation (SD), and the significance level was set at α = 0.05.

For multiple comparisons, the Sidak method was used to adjust p-values, and all reported p-values are corrected. Additionally, effect sizes were reported: partial eta squared (η_p_²) was used for ANOVA, and Cohen’s d was used for pairwise comparisons.

## Results

### Subjective feelings of tDCS

Overall, after five consecutive days of tDCS, the incidence of adverse reactions among the participants was relatively low, mainly manifested as itching and tingling sensations. Moreover, there was no statistically significant difference in subjective feelings between the sham group and the tDCS group (**Table 2**). This indicates that the participants’ subjective sensory experiences of the stimulation were similar between the two groups, which is consistent with the non-significant difference in their Likert scale ratings of stimulation type.

**Table 2:** Subjective feelings of participants.

Symptoms	tDCS (n = 17)	Sham (n = 15)	χ² value	*P* value
Headache n (%)	1 (5.9)	1 (6.6)	0.208	0.867
Itching n (%)	4 (23.5)	4 (26.7)	0.097	0.755
Tingling n (%)	6 (35.3)	4 (26.7)	0.643	0.423
Burning n (%)	2 (11.7)	3 (20.0)	0.507	0.477

### Effects of repetitive tDCS on lower limb knee joint muscle strength

**Figure 1:**
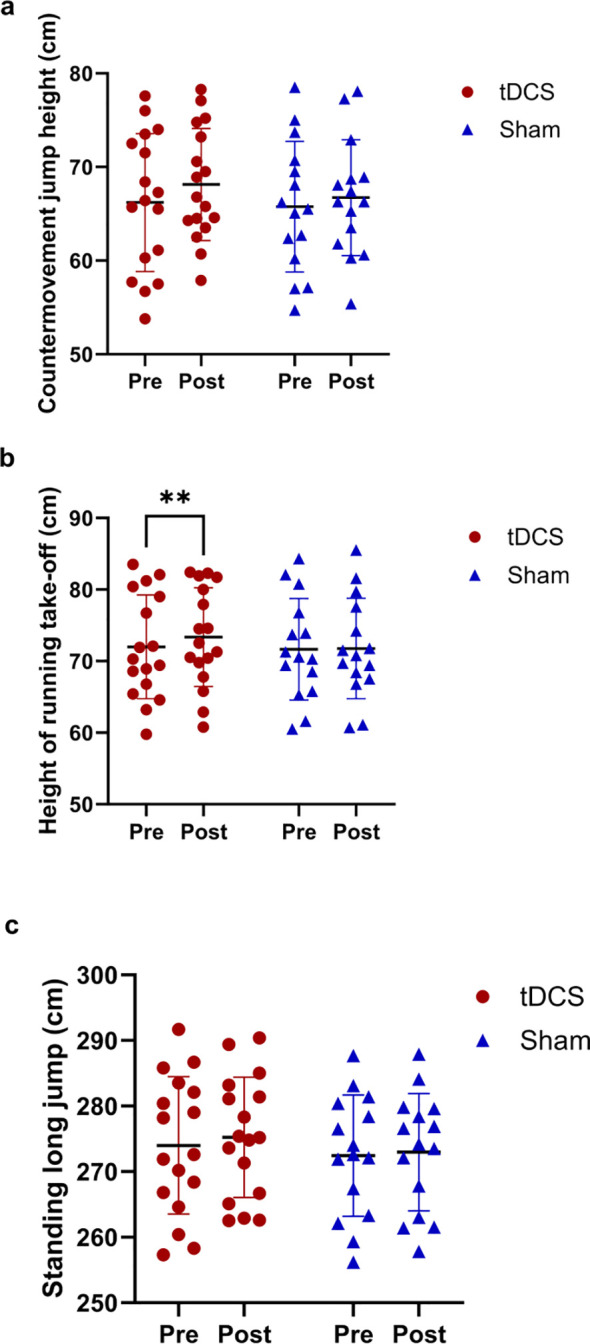
Changes in CMJ, running jump height, and standing long jump of participants before and after experimental intervention. **(a)** represents CMJ, **(b)** represents Running Jump, and **(c)** represents standing long jump. ** indicates that when compared between Pre and Post, *P* < 0.01.

**Figure 1** shows the changes in the countermovement Jump (CMJ), running jump height, and standing long jump distance of the participants before and after the experimental intervention.

Regarding the CMJ, a significant main effect of Time was observed (F (1, 30) = 6.463, *P* = 0.0164, η_p_² = 0.177), while the Time × Group interaction (F (1, 30) = 0.7084, *P* = 0.4066, η_p_² = 0.023) and main effect of Group (F (1, 30) = 0.1633, *P* = 0.689, η_p_² = 0.005) were non-significant. This indicates an overall temporal improvement across both groups, consistent with practice or training adaptation effects.

For the running jump height, a significant Time × Group interaction (F (1, 30) = 5.931, *P* = 0.021, η_p_² = 0.165) and main effect of Time (F (1, 30) = 7.801, *P* = 0.009, η_p_² = 0.206) were detected. *Post-hoc* analysis confirmed a significant increase in the tDCS group (*P* = 0.0013, d = 0.9263), with no significant change in the sham group (*P* = 0.417, d = 0.22), indicating a tDCS-specific enhancement effect on running jump height.

For the standing long jump, no significant main effects of Time (F (1, 30) = 3.108, *P* = 0.0881, η_p_² = 0.094) or Group (F (1, 30) = 0.3303, *P* = 0.5697, η_p_² = 0.011), or interaction effect (F (1, 30) = 0.4641, *P* = 0.5009, η_p_² = 0.015) were found.

**Figure 2:**
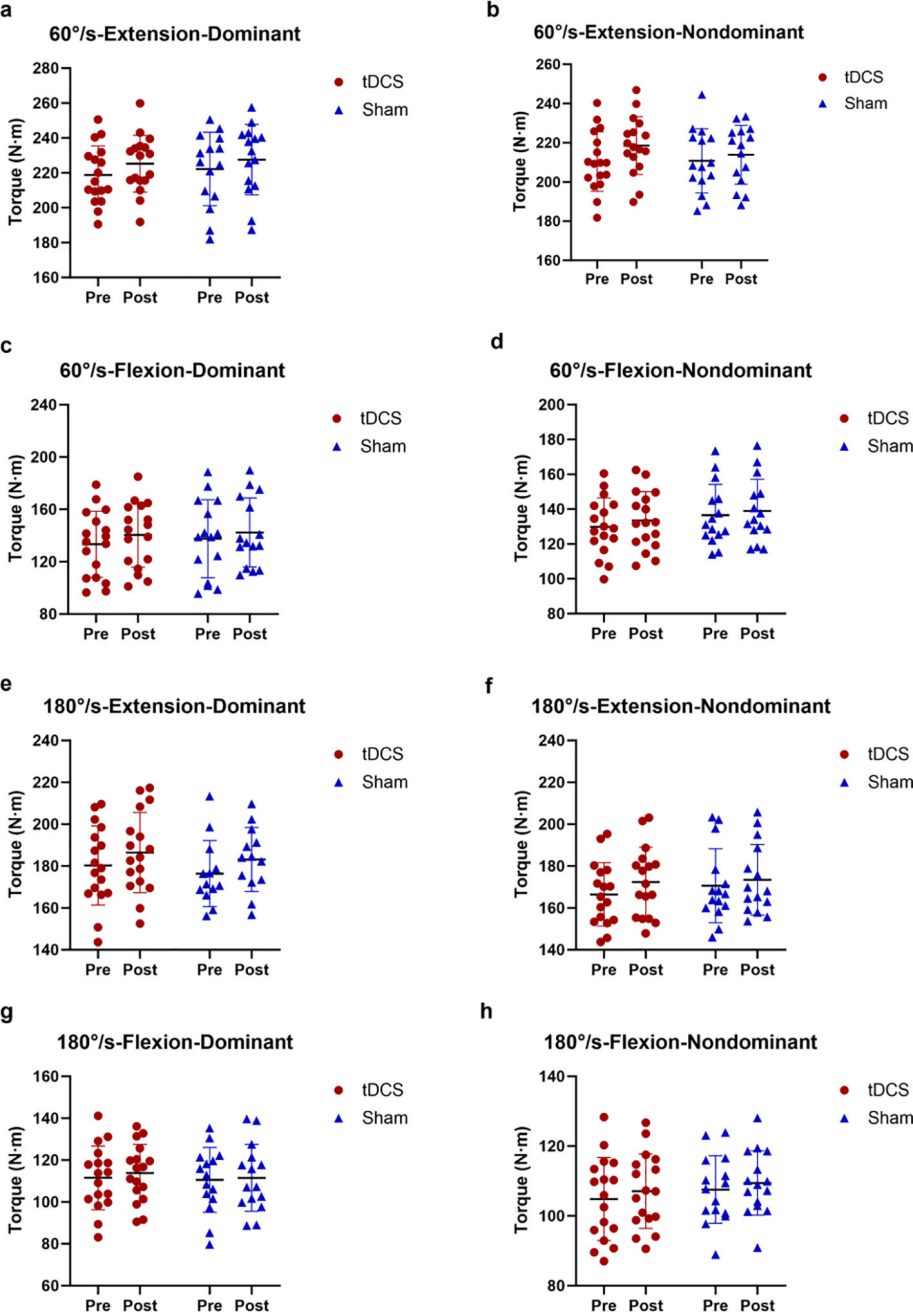
Changes in isokinetic muscle strength of the knee joint at different speeds before and after experimental intervention. **(a)** represents 60°/s-extension-dominant leg; **(b)** represents 60°/s-extension-nondominant leg; **(c)** represents 60°/s-flexion-dominant leg; **(d)** represents 60°/s- flexion-nondominant leg; **(e)** represents 180°/s-extension-dominant leg; **(f)** represents 180°/s- extension-nondominant leg; **(g)** represents 180°/s-flexion-dominant leg; **(h)** represents 180°/s- flexion-nondominant leg.

**Figure 2** presents the changes in isokinetic knee joint muscle strength at different velocities before and after the experimental intervention.

At 60°/s: (1) Dominant leg extension: A significant main effect of Time was observed (F (1, 30) = 12.93, *P* = 0.0011, η_p_² = 0.301), with no significant main effect of Group (F (1, 30) = 0.2065, *P* = 0.6528, η_p_² = 0.004) or Time × Group interaction (F (1, 30) = 0.1129, *P* = 0.7392, η_p_² = 0.004). This reflects a general temporal improvement in muscle strength across both groups. (2) Non-dominant leg extension: A significant main effect of Time was detected (F (1, 30) = 8.384, *P* = 0.0070, η_p_² = 0.218), with no significant group (F (1, 30) = 0.1830, *P* = 0.6718, η_p_² = 0.006) or interaction (F (1, 30) = 1.732, *P* = 0.1982, η_p_² = 0.055) effect, indicating an overall time-related enhancement. (3) Dominant leg flexion: A significant main effect of Time was found (F (1, 30) = 12.35, *P* = 0.0014, η_p_² = 0.292), without significant group (F (1, 30) = 0.1118, *P* = 0.7405, η_p_² = 0.004) or interaction (F (1, 30) = 0.5005, *P* = 0.4847, η_p_² = 0.016) effect. (4) Non-dominant leg flexion: A significant main effect of Time was observed (F (1, 30) = 18.44, *P* = 0.0002, η_p_² = 0.381), with no significant group (F (1, 30) = 1.009, *P* = 0.3232, η_p_² = 0.033) or interaction (F (1, 30) = 0.5953, *P* = 0.4464, η_p_² = 0.019) effect, reflecting a common temporal adaptation in both groups.

At 180°/s: (1) Dominant leg extension: A significant main effect of Time was observed (F (1, 30) = 12.01, *P* = 0.0017, η_p_² = 0.300), with no significant main effect of Group (F (1, 30) = 0.3332, *P* = 0.5684, η_p_² = 0.011) or Time × Group interaction (F (1, 30) = 0.0223, *P* = 0.8822, η_p_² = 0.0007). This reflects a general temporal improvement in isokinetic muscle strength across both groups. (2) Non-dominant leg extension: A significant main effect of Time was detected (F (1, 30) = 12.33, *P* = 0.0014, η_p_² = 0.291), without significant group (F (1, 30) = 0.2045, *P* = 0.6544, η_p_² = 0.007) or interaction (F (1, 30) = 1.481, *P* = 0.2331, η_p_² = 0.047) effect, indicating an overall time-related enhancement in both groups. (3) Dominant leg flexion: No significant main effects of Time (F (1, 30) = 2.516, *P* = 0.1232, η_p_² = 0.077) or Group (F (1, 30) = 0.0999, *P* = 0.7541, η_p_² = 0.003), or Time × Group interaction (F (1, 30) = 0.4277, *P* = 0.5181, η_p_² = 0.014) were found, suggesting no notable temporal changes in muscle strength for this subcondition. (4) Non-dominant leg flexion: A significant main effect of Time was observed (F (1, 30) = 8.667, *P* = 0.0062, η_p_² = 0.224), with no significant group (F (1, 30) = 0.4796, *P* = 0.4939, η_p_² = 0.016) or interaction (F (1, 30) = 0.0620, *P* = 0.8051, η_p_² = 0.002) effect, consistent with common temporal adaptation across participants.

### Effects of repetitive tDCS on the cognitive function

#### Reaction time

**Figure 3:**
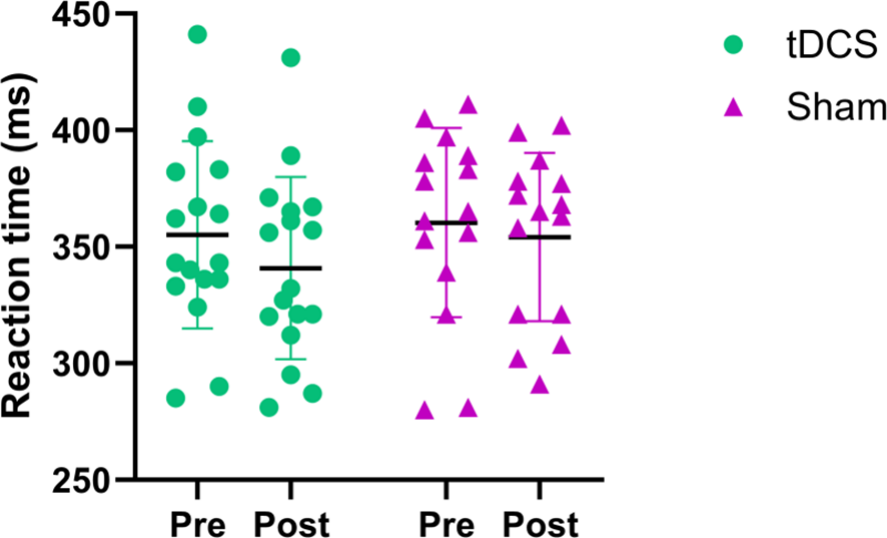
Changes in reaction time before and after experimental intervention.

**Figure 3** shows the changes in reaction time (RT) of the two groups of participants before and after the experimental intervention. Regarding the RT, a significant main effect of Time was found (F (1, 30) = 14.54, *P* = 0.0006, η_p_² = 0.326), with non-significant Time × Group interaction (F (1, 30) = 2.268, *P* = 0.1425, η_p_² = 0.070) and main effect of Group (F (1, 30) = 0.4719, *P* = 0.4974, η_p_² = 0.015). This reflects a general practice-related reduction in RT across both the tDCS and sham groups, with no evidence of a tDCS-specific modulation effect on reaction time.

#### No-Go P3 component

**Figure 4:**
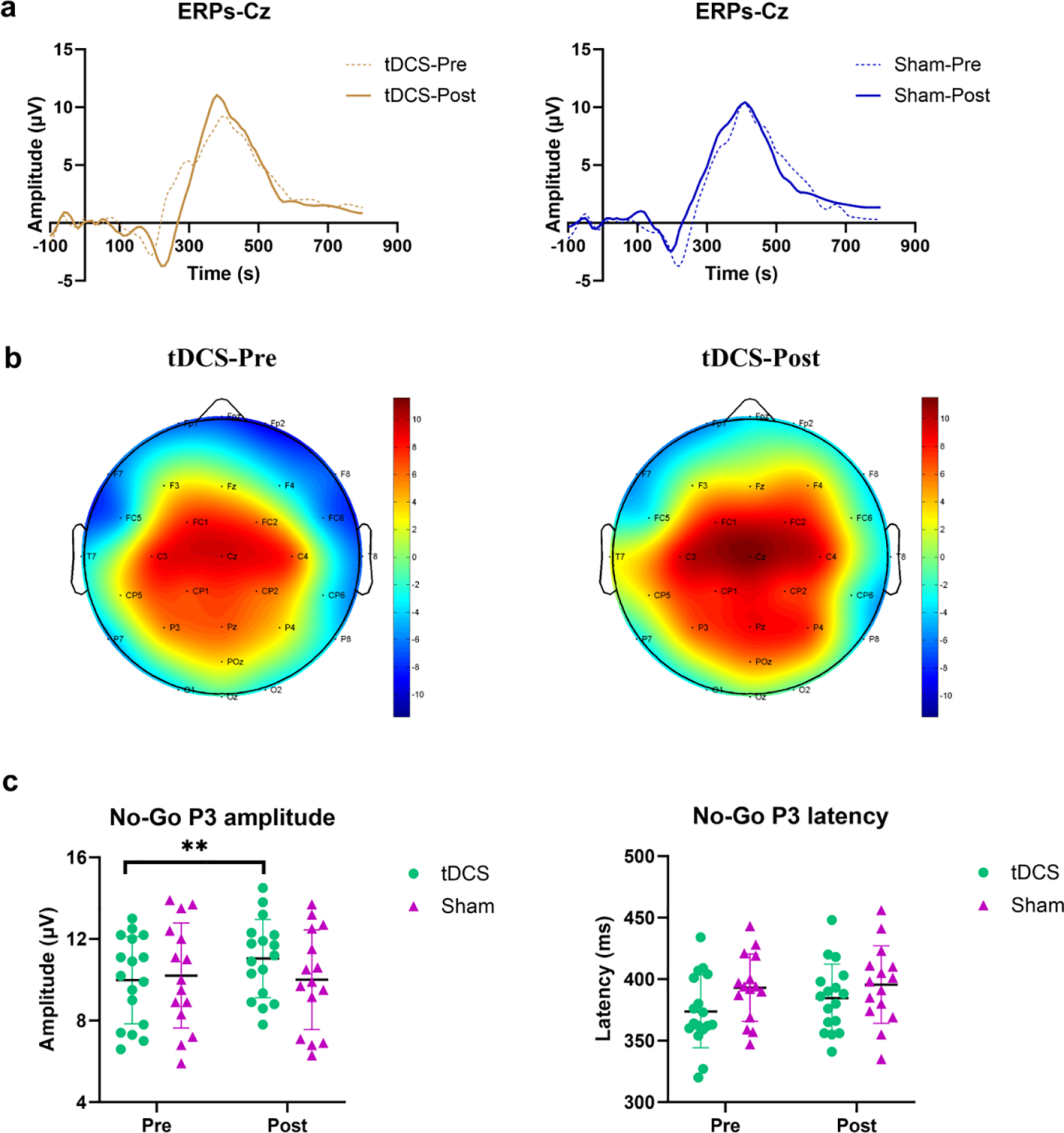
Changes in the amplitudes and latencies of No-Go P3 components at the Cz electrode position evoked by the Go/No-Go paradigm in participants before and after experimental intervention. **(a)** represents the ERPs curve; **(b)** represents the topographies of the No-Go P3 component; **(c)** represents the statistical comparison of No-Go P3 amplitude and No-Go P3 latency. ** indicates that when compared between Pre and Post, *P* < 0.01.

This study used the Go/No-Go paradigm to evaluate the inhibitory control function of the participants. For No-Go P3 amplitude as shown in **Figures 4a, b**, a significant main effect of Time (F (1, 30) = 6.155, *P* = 0.0189, η_p_² = 0.170) and interaction (F (1, 30) = 13.35, *P* = 0.001, η_p_² = 0.308) were observed, but no significant group effect (F (1, 30) = 0.2698, *P* = 0.6073, η_p_² = 0.009). The *post-hoc* demonstrated that tDCS group exhibited a significant post-intervention increase (*P* = 0.0001, d = 1.09), while the sham group did not (*P* = 0.427, d = 0.21). This indicates a greater improvement in inhibitory control in the tDCS group.

As shown in **Figure 4c**, the experimental intervention had no significant main effects of Time (F (1, 30) = 3.024, *P* = 0.0923, η_p_² = 0.0915) or Group (F (1, 30) = 2.508, *P* = 0.1238, η_p_² = 0.077), or interaction effect F (1, 30) = 1.196, *P* = 0.2829, η_p_² = 0.0383) on the No-Go P3 latency of the participants.

## Discussions

This study investigated the effects of five consecutive days of anodal tDCS over the primary motor cortex (M1) on lower limb explosive strength and executive function in male collegiate volleyball players using a randomized single-blind sham-controlled design. The results confirm tDCS-specific improvements in running jump height and No-Go P3 amplitude (significant Time × Group interaction). For other outcomes (CMJ, isokinetic knee strength, reaction time), a significant main effect of Time was observed without a significant Time × Group interaction, indicating general temporal or practice-related improvements across both groups, rather than tDCS-specific modulation. No significant group or interaction effects were found in No-Go P3 latency.

### Subjective feelings of tDCS

During the experimental process of this study, a small number of participants reported mild physical discomfort, but no serious adverse reactions occurred. Based on the subjective feelings collected from the participants after receiving tDCS, all participants had good tolerance to the tDCS stimulation and did not report any serious adverse reactions. Only mild itching, tingling, and burning sensations occurred during the current rise and fall. Moreover, no delayed adverse reactions were reported after the stimulation ended, which confirms the safety of 5 consecutive days of 2 mA anodal tDCS over M1 for trained volleyball athletes.

### Effects of tDCS on muscle strength

In this study, the choice of the Halo Sport device was driven by its suitability for athletic training environments and alignment with our theoretical target (M1). The device’s preconfigured M1 leg-area stimulation is consistent with the 10–20 system and has been validated in previous sports-related tDCS studies (Codella et al., 2021; Lu et al., 2021). While the device limits montage flexibility, M1 was prioritized as the target due to its direct role in lower-limb motor control and documented efficacy for enhancing explosive power in trained populations (Lattari et al., 2020; Kamali et al., 2019). The significant main effect of Time for CMJ and isokinetic knee strength (both 60°/s and 180°/s, covering extension/flexion of dominant and non-dominant legs) indicates general improvements in lower limb explosive power across both groups, likely driven by combined training and practice effects. Only the running jump height showed a tDCS-specific enhancement (significant Time × Group interaction, with a significant pre-to-post increase in the tDCS group but no significant change in the sham group), which is highly consistent with volleyball-specific offensive and defensive movement patterns (e.g., spiking, blocking with approach run) and reflects the targeted improvement effect of repeated tDCS on sport-specific explosive jumping ability. The study by Lattari et al. (2020) demonstrated that a single session of anodal tDCS could significantly increase the height of the CMJ and the peak muscle power, which supports the potential of tDCS in improving lower limb explosive power. In this study, CMJ only showed a time-dependent general improvement, which may be related to the different movement characteristics between CMJ (static takeoff) and running jump (dynamic takeoff), and the latter is more dependent on the corticospinal tract excitability regulated by tDCS (Lattari et al., 2020). The significant main effect of Time for CMJ, in the absence of a Time × Group interaction, suggests that the observed improvement was largely driven by practice or training adaptation rather than a tDCS-specific effect. While the tDCS group exhibited a medium effect size (d = 0.599) for its pre-post improvement, the lack of a significant interaction with the sham group indicates that this change cannot be confidently attributed to the stimulation. This contrasts with findings from Lattari et al. (2020) who reported a tDCS-specific effect on CMJ in strength trainers (d = 0.72). This discrepancy may stem from differences in participant populations (strength trainers vs. volleyball players), the dynamics of the jumping task, or the critical distinction between single and repeated stimulation protocols. Our findings contrast with Hadi Fahrvandi et al. (2023), who observed no acute tDCS effects on jumping performance. This discrepancy not only arises from repeated vs. single-session stimulation, highlighting the importance of cumulative neural adaptation, but also may be due to the difference in research objects (non-elite jumpers vs. trained male collegiate volleyball players) and the matching degree of jumping tasks with sport-specific characteristics. Klomjai et al. (2018) investigated the effect of a single-session tDCS on the lower limb function of stroke patients and found that tDCS could improve the sit-to-stand performance, but had limited improvement in muscle strength. This also indicates that the effect of a single-session tDCS may not be as significant as multiple stimulations, which also supports the effectiveness of using stimulations of multiple sessions in this study. Addtionally, the lack of significant improvement in standing long jump, despite enhancements in running jump, stems primarily from poor alignment between the test task and volleyball-specific training patterns (Wilkosz et al., 2021), which modulates the efficacy of tDCS-induced neuromodulation.

In this study, the participants were at the level of second-class volleyball players and had certain training experience. They found that anodal tDCS significantly enhanced lower-limb explosive power and muscle strength. For example, Jung et al. (2023) found that the effect of tDCS in healthy adults was not significant, possibly because these participants lacked a sufficient basic training level.

In this study, the participants were second-level volleyball players with a certain amount of training experience. The significant improvement in strength and explosive power among these second-level volleyball players in our study is consistent with the findings of Lattari et al. (2020) on experienced strength trainers. They discovered that anodal tDCS significantly enhanced lower limb explosive power and muscle strength (Lattari et al., 2020). In contrast, beginners and non-athletes have a relatively limited response to tDCS. For instance, Jung et al. (2023) found that the effect of tDCS in healthy adults was not significant, probably due to the insufficient basic training level of these participants.

In addition, this study employed five consecutive days of tDCS combined with exercise training, which contrasts with some studies that only used single session tDCS stimulation. Hendy and Kidgell (2013) found that single session tDCS stimulation had limited impact on muscle strength improvement, while multiple repeated stimulations could more significantly enhance strength and neural adaptability. Wrightson et al. (2020) conducted research and found that applying a single anodal tDCS of either 1mA or 2mA to the motor cortex of the left cerebral hemisphere in healthy participants did not change the subsequent maximum isometric knee extensor strength. From the above mentioned studies, it can be seen that the effect of tDCS on muscle strength may be related to its dosage. Multiple repeated tDCS stimulations may enhance the excitability of the corticospinal tract through a cumulative effect, thus achieving better results in exercise training. The systematic review by MaChado et al. (2019b) also indicated that the application of multiple anodal tDCS is more effective in improving muscle strength compared to single session stimulation.

One of the important mechanisms of tDCS is to increase the excitability of the corticospinal tract, thereby improving muscle recruitment strategies (MaChado et al., 2019b). A key mechanism of tDCS is the enhancement of corticospinal tract excitability, which optimizes muscle recruitment strategies and improves force output (MaChado et al., 2019b). As a non-invasive neuroelectrical stimulation technique, tDCS can regulate the excitability of neurons by applying a weak electric current to specific sites, thus influencing the performance of muscle strength and explosive power. Specifically, tDCS may affect the lower limb muscle strength of volleyball players through the following mechanisms. tDCS stimulation may activate motor neurons, prompting more muscle fibers to participate in movement and thus increasing muscle strength and explosive power (MaChado et al., 2019b).

### Impact of tDCS on executive function

This study found that after five consecutive days of tDCS, the reaction time of the participants in completing the Go/No-Go paradigm showed a significant time-dependent improvement, which only reflects the general practice effect of cognitive task training in both groups and provides no evidence for a tDCS-specific modulation effect on reaction time. The core finding of cognitive function is that No-Go P3 amplitude showed a significant Time × Group interaction, with the tDCS group presenting a significant post-intervention increase, while the sham group had no obvious change, which indicates that repeated tDCS over M1 exerts a specific enhancement effect on the inhibitory control function of male collegiate volleyball players. No-Go P3 is the core ERP component reflecting the late stage of response inhibition and cognitive evaluation in the Go/No-Go task, and its amplitude increase is closely related to the enhancement of the fronto-parietal network’s resource allocation and processing efficiency for inhibitory information (Falkenstein, 2006; Polich, 2007). This tDCS-specific improvement may be attributed to the fact that anodal stimulation of M1 increases cortical excitability, and induces synaptic plasticity in the fronto-parietal network associated with inhibitory control, thereby strengthening the brain’s ability to filter irrelevant information and execute response inhibition—an ability that is crucial for volleyball athletes to make rapid and accurate decisions in complex game situations (Alves et al., 2013). In this study, no significant group or interaction effects were found in No-Go P3 latency, which suggests that repeated tDCS over M1 can improve the efficiency of inhibitory control processing (amplitude increase) but cannot shorten the response time of inhibitory control (latency unchanged). In recent years, an increasing number of studies have shown that tDCS has a positive impact on the cognitive function of athletes (Leite et al., 2011; Gold and Ciorciari, 2021; Moezzi et al., 2023). tDCS has demonstrated certain potential in aspects such as reaction time, response speed, and attention (Gold and Ciorciari, 2021). For example, numerous studies have indicated that repeated tDCS interventions can improve the performance of participants in visual attention tasks (Filmer et al., 2017; Lu et al., 2020).

The tDCS intervention has the potential to enhance the attention and reaction speed of volleyball players, and this might be attributed to its neural regulation mechanisms. tDCS is likely to induce synaptic remodeling and neural plasticity within the cerebral cortex, either strengthening or weakening the connections among different neurons. Such remodeling and plasticity are potentially beneficial for improving the efficiency and effectiveness of cognitive functions (Maudrich et al., 2022).

## Conclusions

Repetitive tDCS over M1 can serve as a promising sport-specific adjunct training method for male collegiate volleyball players, with good safety and tolerance. It exhibits confirmed specific benefits for key volleyball-related motor and cognitive outcomes (significant Time × Group interaction). on the motor side, it specifically improves volleyball-specific explosive jumping performance (running jump) which is closely related to spiking and blocking; on the cognitive side, it specifically enhances inhibitory control function (increased No-Go P3 amplitude) which is essential for complex game decision-making. For lower limb muscle strength (CMJ, isokinetic strength), both the tDCS group and the sham group showed significant time-dependent improvements, with no statistical support for a tDCS-specific enhancement effect, as indicated by the non-significant Time × Group interactions. This suggests that the observed gains are largely attributable to general practice and training adaptations during the intervention period. Repeated tDCS over M1 has no significant regulatory effect on the latency of No-Go P3 of ERP components related to inhibitory control, suggesting that its cognitive modulation effect is limited to the processing efficiency of inhibitory control, rather than the response speed of cognitive processing.

### Limitations and further studies

Despite the promising findings, this study has several limitations that warrant consideration. First, the sample size was relatively small (n=32) and restricted to male collegiate volleyball players, which may limit the generalizability of the results to female athletes, other sports populations, or non-athletes. Second, the intervention duration was limited to five consecutive days, and the long-term sustainability of tDCS effects on muscle strength and cognitive performance remains unclear. Third, the study employed a fixed stimulation protocol (2 mA for 20 minutes) targeting primary motor areas; alternative parameters (e.g., different current intensities, longer stimulation durations, or prefrontal cortex targets) might yield distinct outcomes. Fourth, a methodological limitation of this study is the use of randomized stimulus presentation in the Go/No-Go task. This approach did not restrict the number of consecutive No-Go trials, which may have confounded reaction time and ERP data, as extended No-Go sequences can reduce athletes’ motor preparation readiness, while extended consecutive Go sequences may induce response habituation, both of which can alter the neural correlates of inhibitory control and reduce the interpretability of the ERP results. Therefore, the interpretation of the reaction time and No-Go P3 findings in this study is made with appropriate caution, and future research should adopt constrained pseudorandomization (e.g., limiting consecutive No-Go trials to 2–3 times) to better isolate the pure inhibitory control process and improve the validity of the task. Fifth, the assessment of blinding integrity was limited to a 5-point Likert scale for participant subjective rating, and no formal statistical analysis of correct versus incorrect group allocation guesses was conducted; this limits the rigorousness of the blinding effect evaluation, and future studies should implement a formal blinding verification process with clear classification of correct/incorrect guesses.

While short-term tDCS shows promise as a training adjunct for sports requiring explosive power and rapid decision-making (e.g., volleyball), further research is warranted to validate tDCS effects in female athletes, elite/professional players, and other sport-specific cohorts to determine sex- and skill-level dependencies, long-term efficacy, optimize individualized protocols, and elucidate neurophysiological underpinnings via multimodal assessments.

## Data Availability

The raw data supporting the conclusions of this article will be made available by the authors, without undue reservation.

## References

[B1] Alves H. Voss M. W. Boot W. R. Deslandes A. Cossich V. Salles J. I. . (2013). Perceptual-cognitive expertise in elite volleyball players. Front. Psychol. 4. doi: 10.3389/fpsyg.2013.00036. PMID: 23471100 PMC3590639

[B2] Berriel G. P. Cardoso A. S. da Silva E. S. Schons P. Melo O. U. M. Bernardo R. T. . (2024). Relationship between knee extensor and flexor muscle strength and age in professional volleyball players. Sports Biomech. 23, 3577–3588. doi: 10.1080/14763141.2022.2146530. PMID: 36440752

[B3] Chen N. Watanabe K. (2023). Effect of colour-shape associations on visual feature discrimination. Q. J. Exp. Psychol. (2006) 76, 2285–2292. doi: 10.1177/17470218231156432. PMID: 36717547

[B4] Cheng C. H. Tsai H. Y. Cheng H. N. (2019). The effect of age on N2 and P3 components: A meta-analysis of Go/Nogo tasks. Brain Cogn. 135, 103574. doi: 10.1016/j.bandc.2019.05.012. PMID: 31200173

[B5] Chinzara T. T. Buckingham G. Harris D. J. (2022). Transcranial direct current stimulation and sporting performance: A systematic review and meta-analysis of transcranial direct current stimulation effects on physical endurance, muscular strength and visuomotor skills. Eur. J. Neurosci. 55, 468–486. doi: 10.1111/ejn.15540. PMID: 34904303

[B6] Codella R. Alongi R. Filipas L. Luzi L. (2021). Ergogenic effects of bihemispheric transcranial direct current stimulation on fitness: a randomized cross-over trial. Int. J. Sports Med. 42, 66–73. doi: 10.1055/a-1198-8525. PMID: 32781476

[B7] da Silva MaChado D. G. Bikson M. Datta A. Caparelli-Dáquer E. Unal G. Baptista A. F. . (2021). Acute effect of high-definition and conventional tDCS on exercise performance and psychophysiological responses in endurance athletes: a randomized controlled trial. Sci. Rep. 11, 13911. doi: 10.1038/s41598-021-92670-6. PMID: 34230503 PMC8260713

[B8] Falkenstein M. (2006). Inhibition, conflict and the Nogo-N2. Clin. Neurophysiol. 117, 1638–1640. doi: 10.1016/j.clinph.2006.05.002. PMID: 16798078

[B9] Falkenstein M. Hoormann J. Hohnsbein J. (1999). ERP components in Go/Nogo tasks and their relation to inhibition. Acta Psychologica 101, 267–291. doi: 10.1016/s0001-6918(99)00008-6. PMID: 10344188

[B10] Filmer H. L. Lyons M. Mattingley J. B. Dux P. E. (2017). Anodal tDCS applied during multitasking training leads to transferable performance gains. Sci. Rep. 7, 12988. doi: 10.1038/s41598-017-13075-y. PMID: 29021526 PMC5636876

[B11] Formenti D. Trecroci A. Duca M. Cavaggioni L. D’Angelo F. Passi A. . (2021). Differences in inhibitory control and motor fitness in children practicing open and closed skill sports. Sci. Rep. 11, 4033. doi: 10.1038/s41598-021-82698-z. PMID: 33597630 PMC7889632

[B12] Gold J. Ciorciari J. (2021). Impacts of transcranial direct current stimulation on the action observation network and sports anticipation task. J. Sport Exercise Psychol. 43, 310–322. doi: 10.1123/jsep.2020-0109. PMID: 34140423

[B13] Grosprêtre S. Grandperrin Y. Nicolier M. Gimenez P. Vidal C. Tio G. . (2021). Effect of transcranial direct current stimulation on the psychomotor, cognitive, and motor performances of power athletes. Sci. Rep. 11, 9731. doi: 10.1038/s41598-021-89159-7. PMID: 33958679 PMC8102586

[B14] Guirelli A. R. Carvalho C. A. Dos Santos J. M. Ramiro Felicio L. (2021). Relationship between the strength of the hip and knee stabilizer muscles and the Y balance test performance in adolescent volleyball athletes. J. Sports Med. Phys. Fitness 61, 1326–1332. doi: 10.23736/s0022-4707.21.11744-x. PMID: 33480509

[B15] Guo W. Ren J. Wang B. Zhu Q. (2015). Effects of relaxing music on mental fatigue induced by a continuous performance task: Behavioral and ERPs evidence. PloS One 10, e0136446. doi: 10.1371/journal.pone.0136446. PMID: 26305353 PMC4549311

[B16] Fahrvandi H. Hedayati R. Bagheri R. Paknazar F. J. M. E. J. o. R. Studies H. . (2023). Effects of transcranial direct current stimulation on the kinetic parameters of countermovement jump in healthy non-elite athletes. Middle East. J. Rehabil. Health Stud 11, e131627. doi: 10.5812/mejrh-131627. PMID: 41477121

[B17] Hendy A. M. Kidgell D. J. (2013). Anodal tDCS applied during strength training enhances motor cortical plasticity. Med. Sci. Sports Exercise 45, 1721–1729. doi: 10.1249/MSS.0b013e31828d2923. PMID: 23470308

[B18] Huang L. Deng Y. Zheng X. Liu Y. (2019). Transcranial direct current stimulation with Halo Sport enhances repeated sprint cycling and cognitive performance. Front. Physiol. 10. doi: 10.3389/fphys.2019.00118. PMID: 30837893 PMC6383107

[B19] Jung J. Salazar Fajardo J. C. Kim S. Kim B. Oh S. Yoon B. . (2023). Effect of tDCS combined with physical training on physical performance in a healthy population 95, 149–156. doi: 10.1080/02701367.2023.2166894, PMID: 37036388

[B20] Jurcak V. Tsuzuki D. Dan I. (2007). 10/20, 10/10, and 10/5 systems revisited: their validity as relative head-surface-based positioning systems. NeuroImage 34, 1600–1611. doi: 10.1016/j.neuroimage.2006.09.024. PMID: 17207640

[B21] Kakihana W. Suzuki S. (2001). The EMG activity and mechanics of the running jump as a function of takeoff angle. J. Electromyogr. Kinesiol. 11, 365–372. doi: 10.1016/s1050-6411(01)00008-6. PMID: 11595556

[B22] Kamali A. M. Saadi Z. K. Yahyavi S. S. Zarifkar A. Aligholi H. Nami M. (2019). Transcranial direct current stimulation to enhance athletic performance outcome in experienced bodybuilders. PloS One 14, e0220363. doi: 10.1371/journal.pone.0220363. PMID: 31369607 PMC6675286

[B23] Klomjai W. Aneksan B. Pheungphrarattanatrai A. Chantanachai T. Choowong N. Bunleukhet S. . (2018). Effect of single-session dual-tDCS before physical therapy on lower-limb performance in sub-acute stroke patients: A randomized sham-controlled crossover study. Ann. Phys. Rehabil. Med. 61, 286–291. doi: 10.1016/j.rehab.2018.04.005. PMID: 29763676

[B24] Kristiansen M. Thomsen M. J. Nørgaard J. Aaes J. Knudsen D. Voigt M. (2022). The effect of anodal transcranial direct current stimulation on quadriceps maximal voluntary contraction, corticospinal excitability, and voluntary activation levels. J. Strength Conditioning Res. 36, 1540–1547. doi: 10.1519/jsc.0000000000003710. PMID: 33677460

[B25] Lattari E. Campos C. Lamego M. K. Legey S. Neto G. M. Rocha N. B. . (2020). Can transcranial direct current stimulation improve muscle power in individuals with advanced weight-training experience? J. Strength Conditioning Res. 34, 97–103. doi: 10.1519/jsc.0000000000001956. PMID: 28426515

[B26] Leite J. Carvalho S. Fregni F. Gonçalves Ó.F. (2011). Task-specific effects of tDCS-induced cortical excitability changes on cognitive and motor sequence set shifting performance. PloS One 6, e24140. doi: 10.1371/journal.pone.0024140. PMID: 21909415 PMC3164710

[B27] Lu P. Hanson N. J. Wen L. Guo F. Tian X. (2021). Transcranial direct current stimulation enhances muscle strength of non-dominant knee in healthy young males. Front. Physiol. 12. doi: 10.3389/fphys.2021.788719. PMID: 34987418 PMC8721010

[B28] Lu H. Liu Q. Guo Z. Zhou G. Zhang Y. Zhu X. . (2020). Modulation of repeated anodal HD-tDCS on attention in healthy young adults. Front. Psychol. 11. doi: 10.3389/fpsyg.2020.564447. PMID: 33329194 PMC7714753

[B29] MaChado S. Jansen P. Almeida V. Veldema J. (2019b). Is tDCS an adjunct ergogenic resource for improving muscular strength and endurance performance? A systematic review. Front. Psychol. 10. doi: 10.3389/fpsyg.2019.01127. PMID: 31156520 PMC6532530

[B30] MaChado D. Unal G. Andrade S. M. Moreira A. Altimari L. R. Brunoni A. R. . (2019a). Effect of transcranial direct current stimulation on exercise performance: A systematic review and meta-analysis. Brain Stimul 12, 593–605. doi: 10.1016/j.brs.2018.12.227. PMID: 30630690

[B31] Marin-Jimenez N. Perez-Bey A. Cruz-Leon C. Conde-Caveda J. Segura-Jimenez V. Castro-Piñero J. . (2024). Criterion-related validity and reliability of the standing long jump test in adults: The Adult-Fit project. Eur. J. Sport Sci. 24, 1379–1392. doi: 10.1002/ejsc.12182. PMID: 39167610 PMC11369318

[B32] Maudrich T. Ragert P. Perrey S. Kenville R. (2022). Single-session anodal transcranial direct current stimulation to enhance sport-specific performance in athletes: A systematic review and meta-analysis. Brain Stimulation 15, 1517–1529. doi: 10.1016/j.brs.2022.11.007. PMID: 36442774

[B33] Mesquita P. H. C. Franchini E. Romano-Silva M. A. Lage G. M. Albuquerque M. R. (2020). Transcranial direct current stimulation: No effect on aerobic performance, heart rate, or rating of perceived exertion in a progressive taekwondo-specific test. Int. J. Sports Physiol. Perform. 15, 958–963. doi: 10.1123/ijspp.2019-0410. PMID: 32023547

[B34] Moezzi S. Ghoshuni M. Amiri M. (2023). Transcranial direct current stimulation (tDCS) effects on attention enhancement: A preliminary event related potential (ERP) study 42, 8798–8804. doi: 10.1007/s12144-021-02190-9, PMID: 41933263

[B35] Montuori S. D’Aurizio G. Foti F. Liparoti M. Lardone A. Pesoli M. . (2019). Executive functioning profiles in elite volleyball athletes: Preliminary results by a sport-specific task switching protocol. Hum. Mov. Sci. 63, 73–81. doi: 10.1016/j.humov.2018.11.011. PMID: 30503984

[B36] Moreira A. Moscaleski L. MaChado D. Bikson M. Unal G. Bradley P. S. . (2023). Transcranial direct current stimulation during a prolonged cognitive task: the effect on cognitive and shooting performances in professional female basketball players. Ergonomics 66, 492–505. doi: 10.1080/00140139.2022.2096262. PMID: 35766283

[B37] Nitsche M. A. Paulus W. (2000). Excitability changes induced in the human motor cortex by weak transcranial direct current stimulation. J. Physiol. 527 Pt 3, 633–639. doi: 10.1111/j.1469-7793.2000.t01-1-00633.x. PMID: 10990547 PMC2270099

[B38] Nitsche M. A. Paulus W. (2001). Sustained excitability elevations induced by transcranial DC motor cortex stimulation in humans. Neurology 57, 1899–1901. doi: 10.1212/wnl.57.10.1899. PMID: 11723286

[B39] Novita N. Oka Harahap P. Sahputera Sagala R. Natas Pasaribu A. M. (2022). Effect of plyometric exercises on limb muscle power in volleyball players. Jurnal SPORTIF: Jurnal Penelitian Pembelajaran 8, 131–144. doi: 10.29407/js_unpgri.v8i1.17810. PMID: 41875081

[B40] Petrigna L. Amato A. Longo G. Castorina A. Pajaujiene S. Musumeci G. (2025). A standard operating procedure for the evaluation of vertical jumps performance through surface electromyography assessment: A scoping review. J. Electromyogr. Kinesiol. 83, 103028. doi: 10.1016/j.jelekin.2025.103028. PMID: 40561822

[B41] Polich J. (2007). Updating P300: an integrative theory of P3a and P3b. Clin. Neurophysiol. 118, 2128–2148. doi: 10.1016/j.clinph.2007.04.019. PMID: 17573239 PMC2715154

[B42] Polich J. Alexander J. E. Bauer L. O. Kuperman S. Morzorati S. O’Connor S. J. . (1997). P300 topography of amplitude/latency correlations. Brain Topogr. 9, 275–282. doi: 10.1007/bf01464482. PMID: 9217986

[B43] Reis J. Schambra H. M. Cohen L. G. Buch E. R. Fritsch B. Zarahn E. . (2009). Noninvasive cortical stimulation enhances motor skill acquisition over multiple days through an effect on consolidation. Proc. Natl. Acad. Sci. U.S.A. 106, 1590–1595. doi: 10.1073/pnas.0805413106. PMID: 19164589 PMC2635787

[B44] Reitmayer H.-E. Monea D. (2021). Relationship between lower limb power and dynamic stability in volleyball players. Timisoara Phys. Educ. Rehabil. J. 14, 29–33. doi: 10.2478/tperj-2021-0003. PMID: 40909103

[B45] Seidel O. Ragert P. (2019). Effects of transcranial direct current stimulation of primary motor cortex on reaction time and tapping performance: A comparison between athletes and non-athletes. Front. Hum. Neurosci. 13. doi: 10.3389/fnhum.2019.00103. PMID: 31024275 PMC6460944

[B46] Vergallito A. Varoli E. Pisoni A. Mattavelli G. Del Mauro L. Feroldi S. . (2023). State-dependent effectiveness of cathodal transcranial direct current stimulation on cortical excitability. Neuroimage 277, 120242. doi: 10.1016/j.neuroimage.2023.120242. PMID: 37348625

[B47] Wilkosz P. Kabacinski J. Mackala K. Murawa M. Ostarello J. Rzepnicka A. . (2021). Isokinetic and isometric assessment of the knee joint extensors and flexors of professional volleyball players. J. Int. J. Environ. Res. 18, 6780. doi: 10.3390/ijerph18136780. PMID: 34202540 PMC8297237

[B48] Wrightson J. G. Twomey R. Yeung S. T. Y. Millet G. Y. (2020). No effect of tDCS of the primary motor cortex on isometric exercise performance or perceived fatigue. Eur. J. Neurosci. 52, 2905–2914. doi: 10.1111/ejn.14651. PMID: 31846516

[B49] Zhang N. An W. Yu Y. Wu J. Yang J. (2024). Go/No-Go ratios modulate inhibition-related brain activity: An event-related potential study. Brain Sci. 14, 414. doi: 10.3390/brainsci14050414. PMID: 38790393 PMC11117662

[B50] Zhang X. Zhang J. Nitsche M. A. Yue T. Guo F. Qi F. (2025). Intensity-dependent transcranial direct current stimulation effects on lower limb strength: optimizing acute and prolonged gains in explosive force and maximal strength. Neuroscience 583, 136–145. doi: 10.1016/j.neuroscience.2025.08.002. PMID: 40774619

